# Strengthening diagnostic capacity in Africa as a key pillar of public health and pandemic preparedness

**DOI:** 10.1371/journal.pgph.0001998

**Published:** 2023-06-13

**Authors:** Kenji O. Mfuh, Ngu Njei Abanda, Boghuma K. Titanji

**Affiliations:** 1 Department of Anatomic Pathology and Clinical Laboratories, Stanford Medicine, Palo Alto, California, United States of America; 2 Department of Virology, Centre Pasteur of Cameroon, Yaoundé, Cameroon; 3 Division of Infectious Diseases, Emory University School of Medicine, Atlanta, Georgia, United States of America; PLOS Global Public Health and APHRC, KENYA

## Introduction

According to recent forecasts from the United Nation’s Population Division, Africa’s population is expected to double from one billion to two billion inhabitants by 2050 [1]. This population growth, combined with climate change and increasing human encroachment on animal habitats, is likely to lead to more outbreaks of emerging diseases [2]–some of which we have seen over the past decade. The Ebola outbreak that occurred in West Africa between 2014–2016 was the most extensive outbreak ever recorded, resulting in the deaths of 11,325 people [3], and since 2020, there have been four outbreaks of the Marburg virus (in Tanzania, Guinea, Equatorial Guinea, and Ghana) and three outbreaks of Ebola virus disease (in the Democratic Republic of Congo and Uganda). These outbreaks emphasize the urgency of strengthening laboratory and diagnostic capacity in Africa so that the continent can become self-sustaining in its ability to respond to such threats.

## Challenges to expanding diagnostic capacity in Africa

Though significant efforts have been made to enhance laboratory capacity in response to the goals outlined in the 2005 International Health Regulation and the Global Health Security Agenda, challenges persist [4]. Laboratory and diagnostic programs in Africa are dominated by external partners and sponsors like the Bill and Melinda Gates Foundation (BMGF), Wellcome, the US government and others [5, 6]. There is often lack of coordination amongst external and national partners with investments channeled through projects which can be duplicative without added benefit and often not aligned with government priorities. For example, during the COVID-19 there have been several similar training programs on next generation sequencing and bioinformatics organized by different agencies, such as WHO, US CDC, APHL, Pasteur, and Africa CDC, which lack coordination. This results in some participants getting repeat training on the same techniques instead of training that adds to existing skills.

Purchased or donated laboratory equipment that is costly and difficult to maintain (Flow cytometers, Sequencers, PCR machines), often ends up requiring technicians from outside Africa to service due to the lack of expertise locally. This leads to significant disruptions in laboratory workflows. Additionally, the implementation of quality management, biosafety, and biosecurity programs is severely underfunded. In situations of outbreak response, existing laboratories are easily overwhelmed with the surge in demand and not equipped to ensure the safety of laboratory personnel.

How do we overcome these challenges to build a truly responsive, resilient laboratory and diagnostic infrastructure that is able to respond to public health needs and emergencies? African governments must step up and take more active roles in these initiatives [7]. They need to define priorities that reflect local needs and prioritize external support towards achieving set goals.

## Areas of growth and opportunity

**Fig 1:**
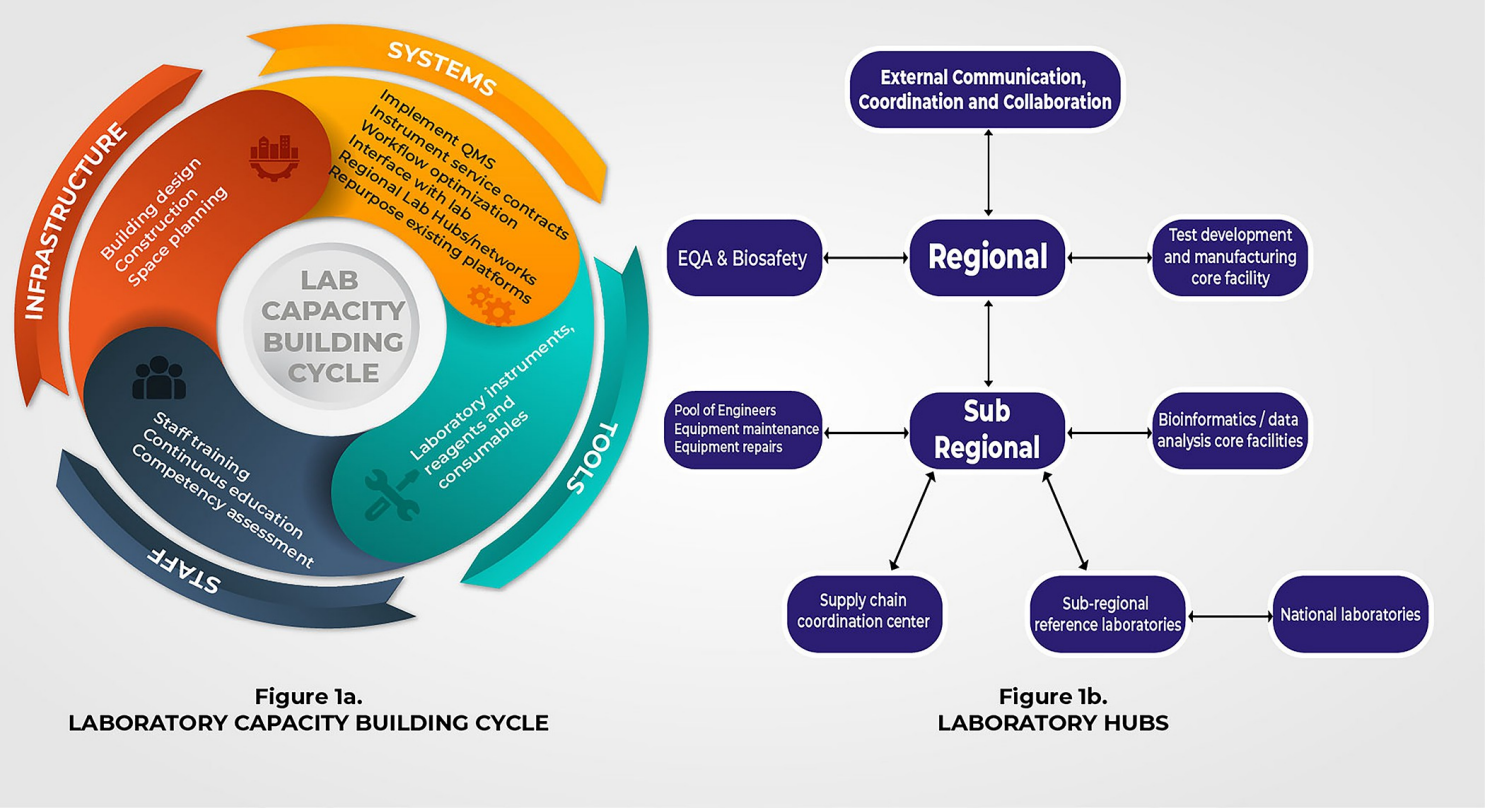
Regional and national approaches to strengthening laboratory diagnostic capacity in Africa. a) Laboratory capacity building cycle b): Inter-regional hubs.

Strengthening laboratory capacity in Africa requires a comprehensive and sustainable strategy that extends beyond crisis response. To achieve this goal, we must prioritize certain areas that offer growth opportunities and serve as a roadmap for success (see Fig 1A).

### Rapid diagnostic testing and portable technologies

Rapid diagnostic testing for Malaria, HIV, Tuberculosis (TB), and COVID-19 have been a success story in Africa [8]. Rapid point of care and self-administered testing kits for HIV which can deliver a result within minutes have reduced costs in testing and improved linkage to HIV care [9, 10]. Similarly for TB, the widespread implementation of rapid molecular testing which is recommended by the World Health Organization, has led to reduced delays in diagnosis and treatment initiation [11]. There is an opportunity to build on these achievements to develop rapid diagnostic tests for other pathogens with pandemic and outbreak potential–indeed, to support COVID-19 diagnostic testing, countries employed existing HIV and TB molecular testing platforms. Just as they were repurposed to respond to the COVID-19 pandemic, these platforms can be adapted proactively for future disease outbreaks by using multiplex approaches capable of diagnosing multiple pathogens. In addition, portable technologies e.g., suitcase lab [12] and MinION (Oxford Nanopore), that have been successfully implemented for diagnosing and sequencing SARS-CoV-2 can be used for other emerging pathogens [13]. Combining these new portable technologies with modular biocontainment facilities makes on-site diagnosis feasible, even in remote locations where outbreaks may occur.

An approach that focuses investment towards expanding rapid testing and molecular diagnostic tools is important for improving scalability and response times in resource constrained settings compared to centralized physical laboratory structures which are more expensive to build and maintain. The more traditional laboratory model can be reserved for referral laboratory tasks such as confirming diagnoses in the context of new outbreak with more effort being committed to decentralizing laboratory capacity. This will enable the rapid deployment of diagnostic teams when an outbreak is suspected, leading to faster containment and response times.

While most African countries have acquired these portable technologies and are ready to deploy in case of an outbreak [12] a potential limiting factor is the reliance on suppliers outside Africa for reagents and consumables. Having manufacturers in Africa capable of producing these diagnostic tools will boost the scalability and utilization of these technologies. Collaborative partnerships between African research institutions, universities, governments, biotechnology companies, and international organizations can facilitate R&D, technology transfer, and capacity building for local production of diagnostic tools. For example, the Pasteur institute in Senegal recently launched Diatropix a non-profit initiative in collaboration with industrial partners to develop rapid diagnostic tests which can be produced locally in Africa [14]. Encouraging more of such initiatives will provide access to expertise locally, draw more funding, and regulatory support and promote regional knowledge sharing and innovation. All of these factors are key to wean over-reliance on foreign support from outside Africa.

### National and regional laboratory networks

Creating national laboratory networks which communicate with regional hubs of excellence across Africa will enhance the continent’s laboratory capacity by consolidating resources and facilitating knowledge exchange, and collaboration [15, 16] (Fig 1B). For instance, a national laboratory in a given country could either conduct wet lab genomic sequencing and share its data with a regional bioinformatics hub for analysis or refer positive samples for genomic sequencing and analysis [17]. At the peak of the COVID-19 pandemic, countries referred COVID-19 positive samples to reference sequencing laboratories in Senegal, Nigeria and South Africa for sequence identification [18]. The surveillance of other diseases like measles, yellow fever and polio employ similar sample referral networks [19]. Establishing a bioinformatics hub in Africa will need more investment in infrastructure including high-speed internet, reliable electricity supply, and adequate computing resources. This needs to happen alongside developing and strengthening mechanisms for secure data sharing. Another benefit of regional hubs is having a pool of trained biomedical engineers who can service instruments from different manufacturers, reducing reliance on imported expertise from outside the continent. By ensuring prompt equipment maintenance and reducing downtimes, these hubs can enhance efficiency and minimize delays in diagnosis. Funding to establish and sustain these regional hubs could be provided directly by African governments themselves or indirectly through the regional economic commissions or through the African Union. Additional support could also be provided by developmental agencies and organizations including The Global Fund, The World Bank, and other philanthropic foundations.

### Strengthening infrastructure and regional laboratory supply chains

Relying on local government funding or international partners to build and maintain laboratory infrastructure is not enough. Innovative financing mechanisms through public-private partnerships should be encouraged and incentivized to secure additional funding for laboratory capacity building. This sector has tremendous growth potential and opportunities for manufacturing, training, and job creation. Additionally, improving the supply chain and logistics to ensure that reagents and consumables can be delivered promptly or even manufactured locally is crucial. Establishing efficient workflows with airlines and shipping companies and implementing local regulations that facilitate the shipment of reagents, consumables, equipment, and biological resources and samples will greatly relieve existing logistical barriers [20].

### Training of personnel and skills maintenance

In Africa, it is crucial to prioritize training skills in bioinformatics, data analysis and interpretation, and laboratory techniques. The short-term strategy should focus on hands-on training for laboratory scientists in advanced techniques such as Next Generation Sequencing library preparation, genomic data quality control analysis, specific bioinformatics pipelines, and utilization of open platforms like GISAID, Nextstrain, and Pangolin. Instead of hosting training workshops in western countries, laboratory infrastructure in Africa should be utilized for these. The African CDC’s Pathogen Genomics and Bioinformatics Fellowship Program is an excellent example of a short-term training program, with two tracks covering the wet lab and dry lab techniques. Long-term training programs should be tailored to more advanced skills such as script writing, sequencing data curation, phylogeny tree analysis, primer design, test development, and field service engineer training, all aimed at enhancing the continent’s diagnostic capacity. African governments, in collaboration with international partners, can establish contextually tailored training facilities and programs that meet regional needs instead of relying on external funders to set the agenda for training. Additionally, establishing robust regulatory frameworks at national and regional levels is crucial to ensure high-quality laboratory services. This means developing and implementing national policies and guidelines for laboratory quality assurance, as well as establishing accreditation systems that ensure laboratories and personnel meet pre-established standards.

## Conclusion

Improving and strengthening laboratory capacity is critical for global public health. In order for Africa to achieve the ambitious goal of becoming self-sustaining in this area, African countries need to set their priorities in ways that best address the local needs of the people, invest financially in strengthening this sector and be committed to contributing to and supporting regional hubs. International partners have to be receptive to input from local leaders in prioritizing funding to areas that need the most investment to bridge existing gaps. An approach that has all stake holders working together towards a common goal will ensure the seamless flow of information, knowledge and resources between national and regional hubs. This will lead to stronger partnerships and ultimately better public health outcomes for Africa and the world.
